# Age-dependent immune landscapes affect influenza vaccine effectiveness and guide precision strategies

**DOI:** 10.3389/fimmu.2026.1919049

**Published:** 2026-08-24

**Authors:** Jing Liu, Zhegang Zhang, Jiayou Zhang, Xuanxuan Nian

**Affiliations:** Wuhan Institute of Biological Products Co., Ltd., Wuhan, China

**Keywords:** age-dependent immunity, influenza, next-generation vaccines, precision vaccination, vaccine design, vaccine effectiveness

## Abstract

Influenza vaccine effectiveness exhibits significant heterogeneity across age groups, largely shaped by lifelong immune remodeling. This review adopts a continuous spectrum perspective-spanning immune development, immune maturation, and immunosenescence-to delineate host immune responses to influenza across different life stages. Subsequently, the influence of these age-dependent differences on vaccine effectiveness is discussed. Based on these insights, an age-specific vaccine development strategy is proposed to guide differentiated vaccine optimization approaches for children, adults, and the elderly. This review provides a theoretical basis for transitioning from a “one-dose-fits-all” vaccination model and toward an age-specific precision paradigm.

## 1 Introduction

Influenza is an acute respiratory infectious disease caused by influenza viruses, characterized by high transmissibility, marked seasonality, and continuous antigenic evolution. These characteristics contribute to a persistent global health burden, resulting in considerable morbidity and mortality annually. Clinically, influenza presents with a wide range of manifestations, from mild upper respiratory symptoms (e.g., fever, cough, myalgia, fatigue) to severe complications, including viral pneumonia and non-respiratory manifestations involving the cardiovascular and central nervous systems (1).

**Figure 1:**
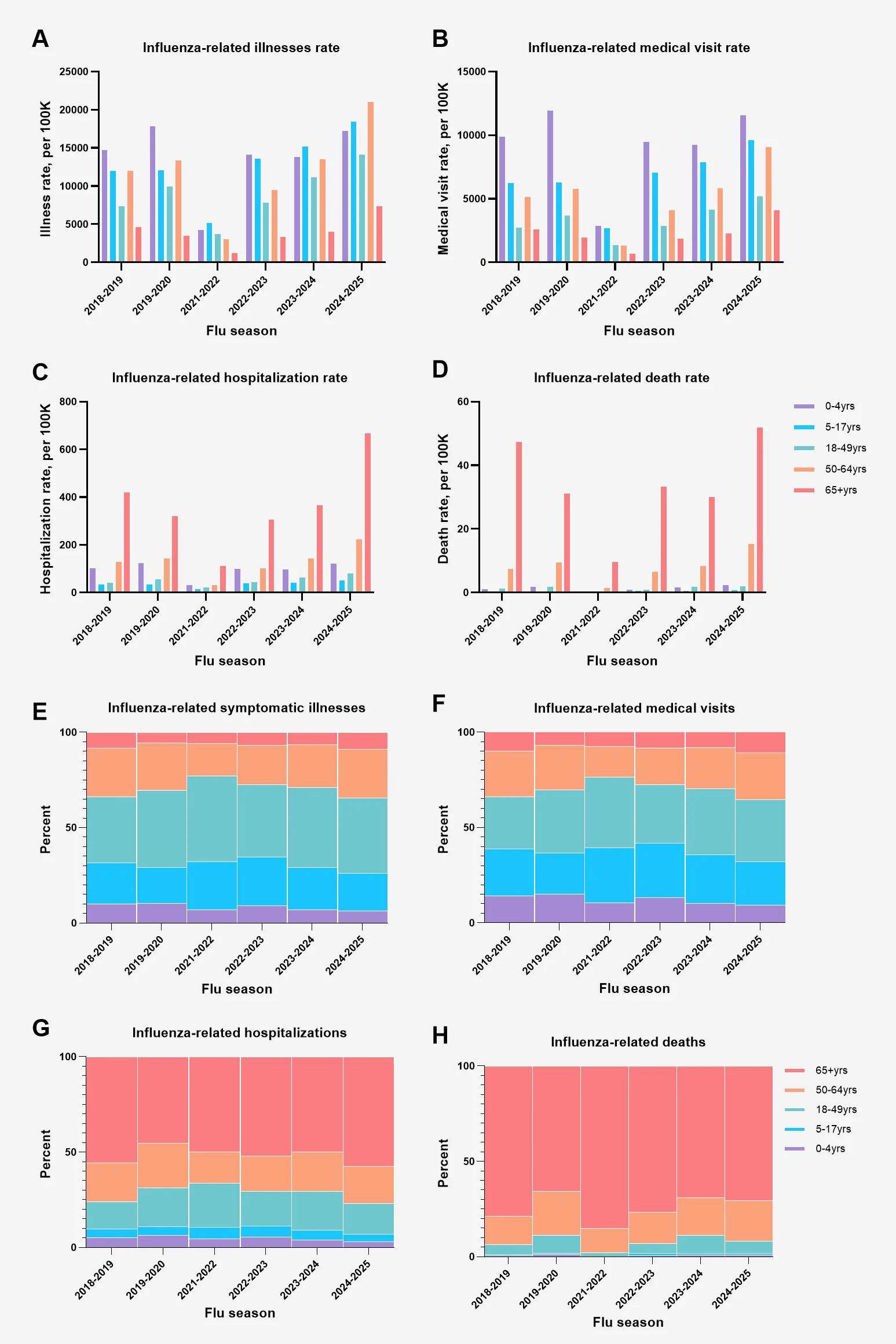
Estimated influenza-related symptomatic illnesses, medical visits, hospitalizations, and deaths during the 2018–2025 influenza seasons. **(A)** Number of influenza-related illnesses per 100,000 population; **(B)** Number of influenza-related medical visits per 100,000 population; **(C)** Number of influenza-related hospitalizations per 100,000 population; **(D)** Number of influenza-related deaths per 100,000 population; **(E)** Proportion of influenza-related symptomatic illnesses by age group; **(F)** Proportion of influenza-related medical visits by age group; **(G)** Proportion of influenza-related hospitalizations by age group; **(H)** Proportion of influenza-related deaths by age group. Data source: U.S. Centers for Disease Control and Prevention (CDC) (4).

Globally, seasonal influenza is estimated to cause approximately one billion infections annually, including 3-5 million severe cases and 290,000–650,000 respiratory deaths (2). The disease burden exhibits pronounced age-dependent heterogeneity. Children under five years of age, particularly those under two, are highly susceptible to severe complications such as pneumonia and influenza-associated encephalopathy (3). Epidemiological data from the U.S. Centers for Disease Control and Prevention (CDC) during the 2018-2025 influenza seasons (**Figure 1**) indicate that children aged 0–4 years had among the highest influenza incidence and outpatient visit rates (**Figures 1A, B**), followed by those aged 5-17 years (**Figure 1D**). Adults aged 18-64 years exhibit lower incidence and medical visit rates than children, and lower hospitalization and mortality rates than older adults (**Figures 1A–D**), but contribute the largest number of influenza-related infections and outpatient visits among all age groups (**Figures 1E, F**). This group also bears the largest indirect economic burden, accounting for approximately 67% of total influenza-associated costs, largely driven by productivity losses (5–7). Within this population, individuals aged 50-64 years represent a transitional high-risk subgroup, characterized by a threefold higher hospitalization rate, prolonged hospital stays, and a markedly increased mortality risk compared with younger adults aged 18–49 years (8, 9). For individuals ≥65 years, reported influenza incidence and outpatient visit rates often appear lower than in children and young adults (**Figures 1A, B**); however, hospitalization and mortality rates are the highest among all age groups (**Figures 1C, D, G, H**) (10). Disease severity in this group is further amplified by prolonged hospitalization, substantial functional decline, and markedly increased years of life lost compared with younger populations (9, 11).

Collectively, influenza exhibits pronounced age-stratified heterogeneity not only in infection risk but also in clinical outcomes. Such differences underscore the necessity of understanding the host immune system across the lifespan and support the development and implementation of age-specific vaccination strategies to optimize disease prevention and reduce the overall public health burden.

## 2 Influenza virus infection and host immune response mechanisms

Influenza viruses are primarily transmitted through respiratory droplets and aerosols expelled by infected individuals during breathing, coughing, or sneezing. Viral entry is mediated by two major surface glycoproteins, hemagglutinin (HA) and neuraminidase (NA), which function coordinately to facilitate infection. HA, a trimeric glycoprotein, initiates infection by binding to terminal sialic acid (SA) residues on respiratory epithelial cells, thereby facilitating viral attachment and entry. NA enzymatically cleaves SA from both host cell surfaces and viral particles, preventing viral aggregation and facilitating the release of progeny virions from infected cells. Additionally, NA facilitates viral access to SA receptors by removing decoy receptors, thereby enhancing viral infection (12). The functional balance between HA receptor-binding activity and NA enzymatic activity is an important determinant of viral fitness, host adaptation, and transmissibility (13).

**Figure 2:**
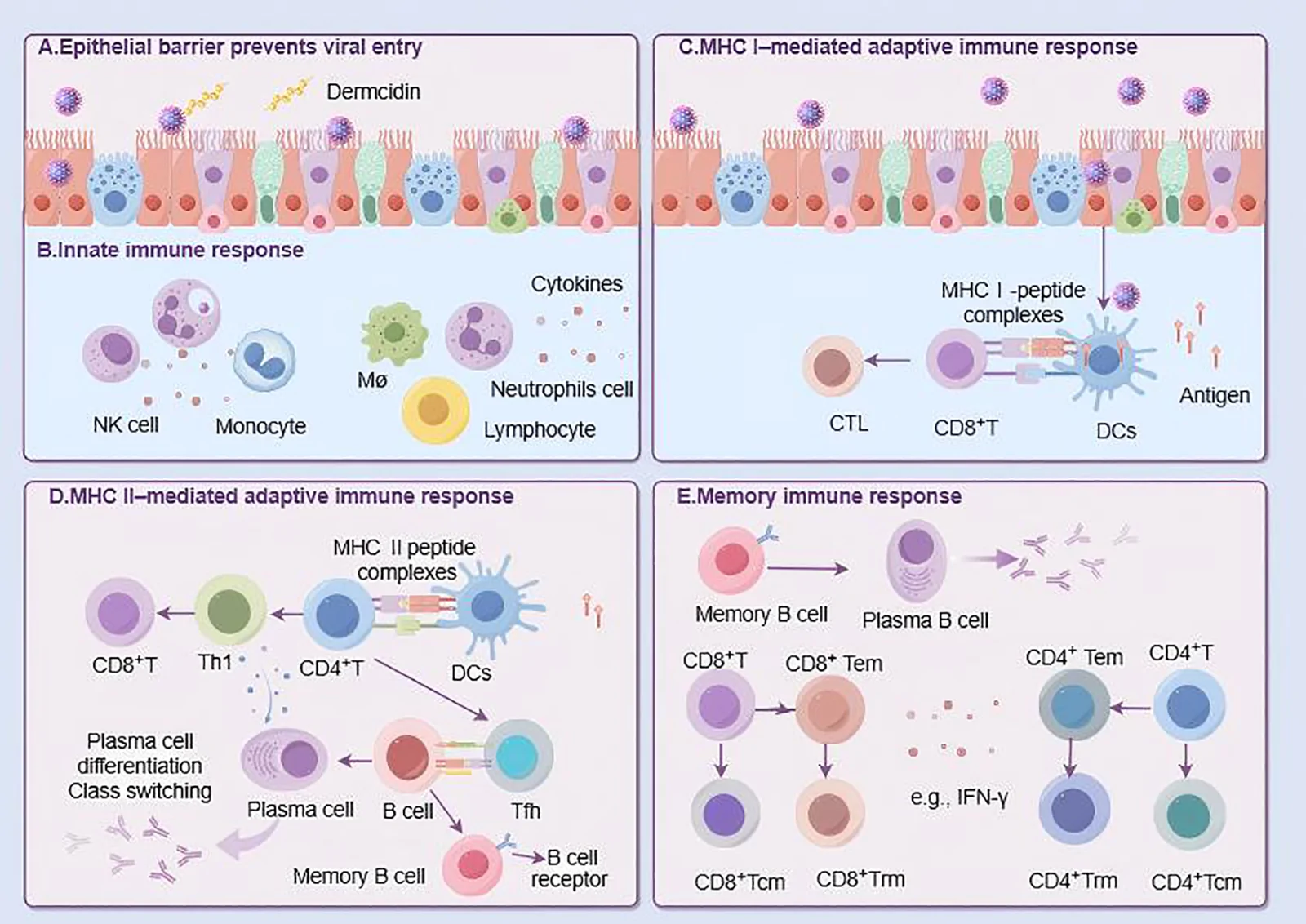
Schematic illustration of host immune response mechanisms to influenza virus infection. **(A)** Mucosal epithelial cells constitute the first physical barrier against viral infection. **(B)** Upon viral invasion, innate immune responses are rapidly activated, triggering interferon production and inflammatory signaling cascades that recruit immune effector cells to the site of infection. **(C)** DCs present viral antigens via MHC I to CD8^+^ T cells, driving their differentiation into CTLs that directly kill infected cells. **(D)** Concurrently, CD4^+^ T cells differentiate into Th1 and Tfh subsets, which coordinate cellular and humoral immune responses by supporting CTL function and facilitating B cell activation and antibody production. **(E)** During and following pathogen clearance, a subset of antigen-specific T cells and B cells differentiates into long-lived memory T cells and memory B cells, establishing durable immunological protection against subsequent reinfection.

Following infection of respiratory epithelial cells, the host mounts a coordinated and multilayered immune response (**Figure 2**). The mucosal immune system (MIS) constitutes the first line of defense, limiting viral attachment and early replication. For instance, dermcidin and its active fragment DCD-1L existing in the nasopharynx and saliva have been reported to interact with conserved regions of HA and exhibit antiviral activity (14). In the upper respiratory tract (URT), mucosal immune responses involve nasal-associated lymphoid tissue (NALT), whereas bronchus-associated lymphoid tissue (BALT) contributes to immune responses in the lower respiratory tract (LRT). Upon viral entry, innate immune responses are rapidly activated through the recognition of viral pathogen-associated molecular patterns (PAMPs) by pattern recognition receptors (PRRs), including Toll-like receptors (TLR3, TLR7, and TLR8), retinoic acid-inducible gene I (RIG-I), and the NLRP3 inflammasome (15). Activation of these pathways induces robust production of type I and type III interferons (IFN-α/β and IFN-λ), as well as pro-inflammatory cytokines and chemokines, establishing an antiviral state (16, 17). This process promotes the recruitment and activation of innate immune effector cells, such as neutrophils, monocytes, and natural killer (NK) cells, which contribute to viral clearance by eliminating infected cells and amplifying local immune responses (18, 19).

Following initial innate immune activation, adaptive immunity is subsequently engaged to provide antigen-specific viral control. Antigen-presenting cells (APCs), particularly dendritic cells (DCs), process and present viral antigens via major histocompatibility complex (MHC) molecules to T cells. Presentation via MHC I molecules activates CD8^+^ T cells, driving their differentiation into cytotoxic T lymphocytes (CTLs) that directly kill infected cells (20, 21). In parallel, antigen presentation via the MHC II pathway activates CD4^+^ T cells, which orchestrate immune responses through functional specialization. CD4^+^ T cells can differentiate into T helper 1 (Th1) cells to assist CD8^+^ T cell–mediated cytotoxicity, or into T follicular helper (Tfh) cells to promote B cell proliferation and differentiation into antibody-secreting plasma cells. Additionally, CD4^+^ T cells enhance the effector functions of innate immune cells, including natural killer (NK) cells and macrophages, by secreting cytokines (22–24).

Following the resolution of infection, a subset of antigen-specific lymphocytes differentiates into long-lived memory cells, establishing the basis for long-term immune protection. Among activated T cells, some persist in the peripheral circulation as central memory T cells (Tcm) and effector memory T cells (Tem), whereas others—specifically a subset of Tem cells—migrate into the lung tissue and differentiate into tissue-resident memory T cells (Trm) (25, 26). Trm cells, particularly those localized in the respiratory mucosa, provide a critical first line of adaptive immunity by mediating rapid and localized immune responses upon re-exposure (27). CD8^+^ Trm cells secrete key inflammatory cytokines such as IFN-γ and TNF-α, promote antiviral states in airway epithelial cells, mediate cytotoxicity, promote DC maturation, and recruit circulating T cells, B cells, and NK cells to rapidly amplify local anti-infective immune responses (28, 29). CD4^+^ Trm cells are multifunctional. They produce cytokines, help recruit and activate B cells, CD8^+^ T cells, and innate cells (30, 31). Meanwhile, memory B cells and long-lived plasma cells sustain humoral immunity by maintaining baseline antibody levels and enabling rapid production of high-affinity antibodies upon reinfection (32, 33). Collectively, these memory compartments contribute to enhanced viral control upon secondary exposure, although their effectiveness may be modulated by antigenic variation and immune imprinting.

## 3 Age-dependent immune landscape

**Figure 3:**
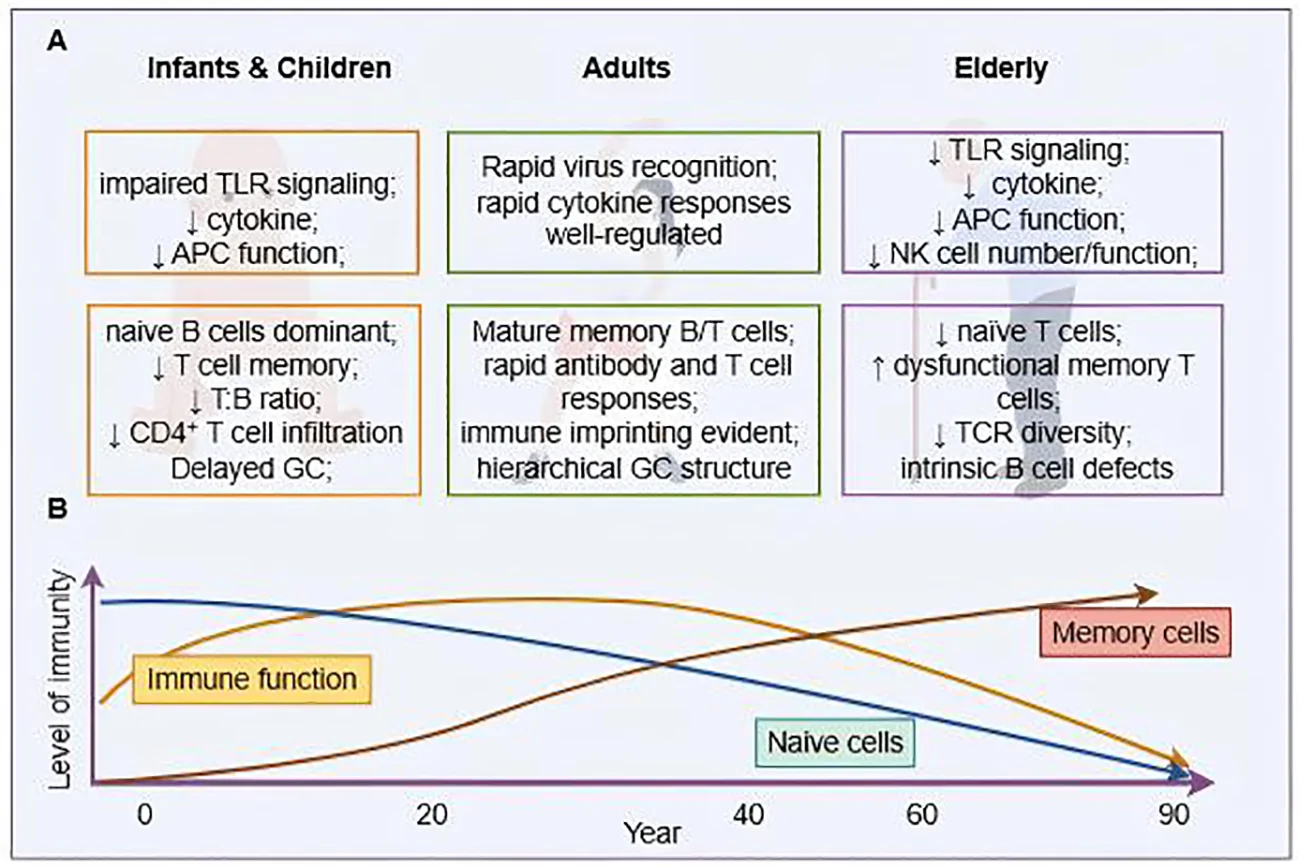
Schematic illustration of age-dependent host immune response characteristics. **(A)** Comparative summary of innate and adaptive immune characteristics across three age groups: infants & children, adults, and the elderly. Detailed features for each age group are presented in the schematic. **(B)** age-related immune function trends. Immune function peaks in adulthood and progressively declines with age. Memory cells increase with age, whereas naïve cells decrease with age.

Age-dependent heterogeneity in immune system is a key determinant of susceptibility to influenza infection, disease severity, and clinical outcomes (**Figure 3**). Throughout life, immune function undergoes continuous remodeling, evolving from a developmentally regulated, tolerance-promoting state in early life to peak immunocompetence during adulthood, before progressively declining into immunosenescence accompanied by chronic low-grade inflammation (inflammaging) in older adults. These distinct immunological landscapes shape antiviral immunity, inflammatory responses, and vaccine responsiveness across the lifespan.

### 3.1 Infants and children

The early-life immune system should not be considered a defective or incomplete version of adult immunity, but rather a developmentally regulated immune state characterized by tolerance-promoting pathways and regulatory immune networks. This regulatory immune landscape facilitates postnatal commensal microbial colonization while limiting excessive inflammation and systemic immunopathology during immune development (34). Although many TLRs are expressed in neonatal immune cells at levels comparable to those in adults, TLR-mediated signaling exhibits a developmentally regulated transcriptional bias, favoring the production of IL-6 and IL-10 while limiting the generation of bioactive IL-12p70 (35, 36). In addition, downstream TLR signaling pathways, including NF-κB- and IRF3/IRF7-dependent signaling networks, display distinct activation and regulatory patterns during early life (37, 38). Compared with adults, neonatal DCs, monocytes, macrophages, and granulocytes display age-specific functional down-regulation (39). For example, neonatal DCs exhibit reduced production of Th1-polarizing cytokines such as IL-12, resulting in impaired induction of IFN-γ-producing T-cell responses (40, 41). CD71^+^ erythroid cells (CECs) further contribute to immune homeostasis by promoting immune tolerance and modulating innate immune activation through the production of regulatory mediators and cytokines. Recent studies have demonstrated that increased frequencies of nucleated erythroid cells in the cord blood of infected neonates are associated with altered immune responses, suggesting their involvement in regulating inflammatory processes during neonatal infection (42). Human bone marrow-derived nucleated erythroid cells have been shown to produce a broad spectrum of cytokines, including IL-1β, IL-2, IL-4, IL-6, IFN-γ, TNF-α, TGF-β, and IL-10, highlighting their capacity to actively shape neonatal immune responses (43).

Adaptive immunity also displays characteristic developmental features. During early life, thymic activity is relatively high, resulting in abundant production of naïve T cells, while the B-cell compartment is likewise dominated by naïve and transitional B cells with relatively few memory B cells because of limited antigen exposure (44). This developmental organization establishes a broad naïve B-cell repertoire that is progressively shaped by microbial colonization and environmental antigen exposure during early life (45, 46). Early-life CD4^+^ T cells exhibit reduced memory differentiation and display an “innate-like” phenotype, consistent with a developmental immune program optimized for regulation and tolerance rather than rapid recall responses (47). Similarly, tissue-resident memory T (Trm) cells are not yet fully established during infancy. Although Trm cells begin to accumulate in mucosal tissues such as the gut and lungs soon after birth, they undergo progressive functional maturation with age, limiting rapid local recall responses to influenza during early life (48, 49). In parallel, germinal center (GC) formation is delayed due to limited expansion of Tfh cells and immature follicular dendritic cells (FDCs), resulting in inefficient affinity maturation and memory B-cell generation (50). Neonates naïve CD4^+^ T cells expressed higher levels of IL-23R, RORγt, and STAT3 prior to activation and exhibit an intrinsic bias toward Th17 differentiation following activation (51). In addition, compared with adults, infants have significantly lower T:B cell ratios in blood, spleen, mesenteric lymph nodes, and intestinal tissues (52). Studies in both mice and humans indicate that CECs regulate adaptive immune responses by limiting excessive B-cell proliferation and antibody secretion, modulating cytotoxic T-cell expansion, and promoting regulatory T-cell (Treg) differentiation (53).

Clinical and pathological observations further support these findings. Postmortem analyses of fatal pediatric influenza cases reveal markedly reduced CD4^+^ T cell infiltration in lung tissues (54). In severe cases, elevated levels of inflammatory cytokines (e.g., IL-6, IL-12, IFN-γ) suggest dysregulated immune activation. Influenza viruses can further exacerbate immune imbalance by interfering with key signaling nodes, including TLR pathways and interferon regulatory factors, thereby promoting viral dissemination, increasing mortality risk, and predisposing to secondary bacterial infections (55, 56).

Evidence from animal models further supports the concept of developmental immune regulation. Compared with adult mice, neonatal mice exhibit delayed and reduced CD4^+^ and CD8^+^ T cell responses following influenza infection, as well as diminished IFN-γ production by NK cells, T cells, and macrophages (57, 58). The increased susceptibility of neonates to perinatal pathogens is partly mediated by CD71^+^ erythroid cells (CECs), which induce arginase 2–dependent immunosuppression. This developmental regulatory pathway promotes tolerance to commensal microbiota while limiting excessive inflammation (59). Collectively, these findings demonstrate that early-life immunity represents a specialized developmental immune state.

### 3.2 Adults

Innate immunity in adults is characterized by rapid and coordinated antiviral responses. PRRs efficiently detect viral PAMPs, leading to prompt activation of antiviral signaling cascades and production of type I interferons and pro-inflammatory cytokines (e.g., IL-1, IL-6, TNF-α) (60). This early response effectively restricts viral replication and establishes an immune microenvironment that supports adaptive immune activation. Moreover, the innate immune responses in adults are generally well-regulated, effectively limiting viral spread while avoiding the immune dysregulation or excessive inflammation often seen in children or the elderly.

Adaptive immunity in adults is supported by a diverse and established humoral and cellular immune memory. Humoral responses, primarily targeting viral surface antigens such as HA and NA, directly block viral entry and neutralize free virions. CD8^+^ and CD4^+^ T cell responses, largely directed against relatively conserved internal antigens (e.g., NP, M1), play critical roles in limiting viral replication, reducing viral load, and promoting viral clearance, while providing a certain degree of heterosubtypic protection (61, 62). Upon re-exposure to influenza virus or vaccination, influenza-specific respiratory CD8^+^ Trm cells, particularly those targeting conserved antigens such as NP, rapidly produce antiviral cytokines and exert cytotoxic activity. CD4^+^ Trm cells contribute to local immune coordination by supporting antiviral T-cell responses and facilitating B-cell activation in the lung (27, 63). As a result, most infections in healthy adults manifest as mild-to-moderate, self-limiting upper respiratory symptoms (64).

However, repeated natural infections or vaccinations lead to the formation of a broad repertoire of cross-reactive memory B cells, accompanied by pronounced immune imprinting (original antigenic sin), where early-life exposure to specific viral strains continues to dominate subsequent antibody responses (65). Recent fate-mapping studies have provided direct mechanistic evidence that serum antibody responses following homologous re-exposure are overwhelmingly derived from the primary cohort of B cells recruited during the initial antigen encounter, a phenomenon termed “primary addiction” (66). During GC reactions upon re-exposure, high-affinity memory B cells preferentially occupy the limited Tfh cell help and antigen resources through clonal competition, establishing a dominance-driven “hierarchical structure” that restricts the participation of naïve B cells targeting new epitopes and limits the updating of the antibody repertoire (65, 67, 68). However, immune imprinting is not exclusively detrimental and may provide beneficial effects under specific circumstances. For example, childhood exposure to influenza viruses within the same HA phylogenetic group has been associated with reduced risk of severe disease caused by later zoonotic influenza viruses, such as H5N1 and H7N9, suggesting that imprinting can contribute to cross-reactive protection through conserved antigen recognition (69). This imprinting effect has been demonstrated at both individual and population levels. For instance, individuals with early-life exposure to H3N2 viruses tends to generate antibody responses to subsequent H1N1 infection that remain biased toward H3-related epitopes. Even cross-reactive responses via the HA stem can influence neutralization efficiency against new strains. Multivalent antigens (e.g., H1N1 and H3N2) can partially mitigate the bias imposed by a single immunization, providing important guidance for vaccine design (70–72). Large-scale seroepidemiological studies, such as analyses of H3N2 strains circulating in southern China from 1968 to 2008, indicate that the first influenza strain encountered during the first decade of life induces the highest levels of neutralizing antibodies, while antibody responses to subsequently encountered strains decline progressively, demonstrating a significant birth-cohort effect of immune imprinting (73). More recent cohort analyses confirmed that birth year remains one of the strongest predictors of influenza antibody landscapes across adulthood, indicating that primary influenza exposure exerts durable effects on population immunity over the lifespan (74). Moreover, NA also exhibits immune imprinting, with an age-dependent distribution pattern, suggesting that the first influenza exposure in childhood can establish long-term immunological bias (71, 75).

Repeated exposures (sequential infections or immunizations) in animal models further support these findings. In both mouse and ferret models, repeated exposure to different influenza virus strains can induce immune imprinting, yet still elicit broadly neutralizing antibody features. With increasing exposure diversity, antibody responses tend to refocus on conserved epitopes, such as the HA stem, enhancing cross-protection (76, 77). The complexity of antigens and the immune system means that different types of immune exposures (infection versus vaccination) can modulate the formation of immune memory in distinct ways. Consequently, upon stimulation with new vaccine antigens, immune imprinting can either enhance or limit the immune response (78). Although immune imprinting has been implicated in reduced vaccine effectiveness (VE) in some contexts (79), it does not equate to immune failure. Imprinting reshapes the quality and specificity of immune responses. Evidence from the vast majority of clinical studies indicates that influenza vaccination remains highly effective in reducing the risk of infection, severe disease, and death (80). Multiple exposures, whether via vaccination or infection, expand the repertoire of memory B cells reactive to both the HA head and stem regions, and broaden the range of neutralization—factors particularly important for protection against “unknown variant strains” (81–83).

### 3.3 Older adults

In older adults, immune function is progressively compromised by immunosenescence and chronic low-grade inflammation (inflammaging), resulting in coordinated defects in innate sensing, thymic output, germinal center (GC) reactions, and adaptive immune memory. Together, these age-associated alterations impair antiviral immunity, reduce responsiveness to influenza vaccination, and increase susceptibility to severe influenza infection (84).

Innate immune responses are characterized by delayed and dysregulated activation with age. Expression and signaling of viral RNA-sensing receptors, such as TLR3, TLR7, and RIG-I in monocytes, macrophages, and DCs,are reduced with age, leading to delayed production of interferons (IFN-α/β) and inflammatory cytokines (IL-6, TNF-α), resulting in inadequate early viral control (85, 86). NK cell numbers in the lungs are reduced and functionally impaired, potentially decreasing viral clearance efficiency (87). Although the proportion of CD56^-^CD16^+^ NK cell subsets increases, these cells exhibit reduced cytotoxicity and diminished cytokine production, indicating impaired antiviral activity (88). Neutrophil chemotaxis and phagocytic capacity are also diminished (89), and DC antigen presentation function is compromised due to reduced expression of co-stimulatory molecules (CD80/CD86), collectively weakening early antiviral defense and T cell priming (90). As a result, delayed innate immune activation permits sustained viral replication and a higher viral burden during the early stage of infection.

In parallel, aging is accompanied by chronic low-grade sterile inflammation (“inflammaging”), characterized by persistently elevated basal levels of pro-inflammatory mediators (91). Chronic inflammation may induce functional reprogramming of innate immunity, characterized by reduced responsiveness to subsequent stimuli, thereby attenuating acute antiviral responses (84, 92). In severe influenza cases, delayed viral clearance superimposed on the pre-existing inflammatory microenvironment triggers dysregulated compensatory inflammation in the late phase of infection, leading to massive cytokine release and immunopathological injury. Excess cytokines further cause epithelial damage, vascular leakage and pulmonary edema, as well as markedly elevated risks of secondary bacterial infections. These events constitute the leading causes of influenza-related mortality among older adults (93).

Adaptive immunity is similarly affected. Thymic involution leads to reduced naïve T cell output and a consequent shift toward an increased proportion of memory T cells in peripheral compartments; however, the functional quality of these memory populations progressively declines with age. Both CD4^+^ and CD8^+^ T cells exhibit impaired clonal expansion, reduced expression of cytotoxic effector molecules such as granzyme and perforin, and diminished TCR diversity, collectively limiting effective antiviral responses (94–97). B cells also exhibit intrinsic functional impairments. Notably, age-dependent downregulation of activation-induced cytidine deaminase (AID) and the E2A transcription factor impairs class-switch recombination (CSR) and somatic hypermutation (SHM) within GCs, resulting in reduced antibody affinity maturation and memory B-cell generation, ultimately leading to diminished magnitude and quality of humoral immune response (98, 99).

Animal studies provide mechanistic support for these observations. In aged mice, macrophages exhibit lower expression of TLRs and impaired production of pro-inflammatory cytokines (e.g., IL-6 and TNF-α) upon stimulation (100). Following influenza infection, aged animals display delayed recruitment and activation of immune cells, characterized by impaired accumulation and activation of APCs, reduced infiltration and activation of NK cells and T cells, delayed B-cell activation, delayed cytokine and chemokine responses, and exhibited altered inflammatory, memory, and chemotactic profiles, collectively contributing to impaired viral control, increased disease severity, and delayed recover (101, 102). Furthermore, aged mice infected with influenza virus exhibit more severe damage to alveolar epithelial cells (AECs) and delayed regeneration type II AECs, resulting in compromised lung recovery (103).

## 4 Current types of influenza vaccines

Since the introduction of influenza vaccines in the mid-20th century, influenza vaccines have evolved from whole-virus inactivated formulations to split and subunit vaccines. Based on vaccine platforms and antigen formats, licensed influenza vaccines can be broadly categorized into inactivated influenza vaccine (IIV), live attenuated influenza vaccine (LAIV), and recombinant influenza vaccine (RIV) (**Table 1**).

**Table 1:** Mainstream Influenza Vaccines: Types and Representative Products.

Vaccine type	Representative name (brand) - manufacturer	Mechanism of action
Inactivated (IIV) (Split/Subunit) Egg-based	Fluzone (Sanofi) Fluarix (GSK) Afluria (Seqirus) Fluvirin (Seqirus)	Viruses are propagated in fertilized chicken eggs/MDCK cells then inactivated/split. Induces antibodies against HA and NA to prevent viral infection
Inactivated (ccIIV) Cell-based	Flucelvax (Seqirus)	
Recombinant Protein (RIV)	Flublok (Sanofi)	The HA gene is inserted into a baculovirus expression vector, which is used to infect insect cells for large-scale production of HA protein.
Live Attenuated	FluMist (AstraZeneca)	Cold-adapted and temperature-sensitive attenuated live virus sprayed into nostrils. Replicates locally in nasopharynx, activating mucosal (IgA) and systemic immunity.
High-Dose/Adjuvanted	Fluzone HD (Sanofi) Fluad (Seqirus)	Enhances immune response via 4-fold higher antigen dose or adjuvant (MF59). The adjuvant helps more strongly activate antigen-presenting cells.

IIV is the most widely used influenza vaccine globally and is produced via egg-based or cell-based propagation followed by viral inactivation. RIV is produced using insect cell–baculovirus expression systems to generate HA proteins, without relying on viral culture. IIV and RIV primarily elicit humoral immunity. LAIV is administered intranasally and can replicate efficiently at the lower temperatures of the upper respiratory tract but not at the higher temperatures of the lower respiratory tract (104). This unique delivery route enables the induction of mucosal immunity, including secretory IgA responses, as well as activation of tonsillar Tfh cells, B cells, and CD8^+^ T-cell responses. Consequently, LAIV more closely mimics natural infection and may confer broader protection by limiting early viral replication and transmission (105–107). Studies have shown that vaccine-induced cellular immune responses exhibit clear age dependency. Following IIV immunization, children aged 6 months to 4 years show significant increase in influenza A-specific T-cell responses, whereas no significant increases in IFN-γ^+^ T cell or NK cell responses is observed in individuals older than 5 years. After LAIV immunization, children aged 5–9 years demonstrate significant increases in influenza A–specific IFN-γ^+^ CD4^+^ and CD8^+^ T cells, while adults show no significant increase in IFN-γ^+^ T cell or NK cell responses (108). These findings indicate that host age, vaccine type, and prior immune history are key determinants of vaccine-induced immune responses.

## 5 Effectiveness of current influenza vaccines and influencing factors

In the general population, influenza VE typically ranges from 40% to 60%. However, VE is influenced by multiple factors, including variations in circulating viral strains across different seasons and geographic regions (Northern and Southern Hemisphere), vaccine platform and immune response profile, population demographics and baseline health status, as well as prior influenza vaccination history or natural infection experience (109). In addition, vaccine-induced protection exhibits a clear time-dependent decline: multiple studies have shown that VE begins to decrease approximately 3 months after vaccination, with a more pronounced decline observed for the H3N2 subtype. The rate of VE decline is faster in older adults (approximately 10%–11% per month) than in younger adults (approximately 8%–9% per month), indicating that immune durability is significantly influenced by host-age-related factors (110, 111). Different influenza vaccine platforms induce distinct immune response profiles. Unlike IIVs, which primarily induce strain-specific neutralizing antibodies, LAIVs elicit robust lung-localized cellular immune responses, including virus-specific CD4^+^ Trm and CD8^+^ Trm that resemble those generated by natural influenza infection. These vaccine-generated Trm mediated cross-strain protection, independent of circulating T cells and neutralizing antibodies, which persisted long-term after vaccination (112). Therefore, both humoral and cellular immune responses contribute to VE, with neutralizing antibodies providing strain-specific protection and cross-reactive T-cell immunity playing a critical role in limiting disease severity and maintaining protection against antigenically drifted viruses.

### 5.1 Infants and children

Children are a priority population for influenza vaccination. Maternal influenza-specific IgG transferred through the placenta provides passive protection during the first months of life but can transiently attenuate vaccine-induced humoral responses by masking vaccine antigens and limiting *de novo* antibody production (113, 114). In 2012, the World Health Organization (WHO) formally designated children aged 6–59 months as a key target group for influenza vaccine immunization (115). Currently, IIVs remain the primary vaccines used in individuals ≥6 months. For healthy children aged 2-49 years, in countries where licensed, LAIV may be considered when indicated and in the absence of contraindications.

Regarding immunization schedules, optimizing dosing and vaccination regimens is essential. Most currently licensed IIVs are administered as a standardized 0.5 mL dose for individuals aged ≥6 months, reflecting efforts by manufacturers and regulatory authorities, including the U.S. Advisory Committee on Immunization Practices (ACIP), to simplify vaccine administration and reduce dosing errors. However, product-specific exceptions remain for children aged 6–35 months. For example, Afluria and Fluzone have age-specific dosing recommendations, with some formulations allowing a 0.25 mL dose in children aged 6-35 months, depending on the licensed formulation. In contrast, Fluarix and Flucelvax use a 0.5 mL dose throughout their approved pediatric age range. Children who have not previously received two doses of influenza vaccine, or whose vaccination history is unknown, should receive two doses administered at least four weeks apart to ensure adequate protection (116). In very low birth weight or preterm infants (<30 weeks gestationalvery preterm infants (<30 weeks gestational age) or very low birth weight infants, a two-dose IIV regimen has demonstrated robust immunogenicity and acceptable safety, supporting its use in this high-risk subgroup (117).

Studies on the effectiveness of LAIV and IIV in children have yielded variable results, largely attributable to differences in antigenic matching and baseline immune status. A pooled analysis of five U.S. studies (2013–2016) during A/H1N1pdm09 circulation showed that the VE with 95% confidence interval (CI) of IIV against A/H1N1pdm09 was 67% (95% CI: 62, 72), whereas the VE of LAIV was only 20% (95% CI: −6, 39) (118, 119). These findings prompted ACIP to temporarily withdraw its recommendation for LAIV (FluMist) during 2016–2018. However, mathematical modeling suggests that in children without pre-existing immunity to circulating strains, or when encountering novel strains with significant antigenic differences (e.g., antigenic drift or shift), strain-matched LAIV may offer better protection (120). Many clinical studies suggest that LAIV and IIV have comparable effectiveness, or that strain-matched LAIV may confer superior protection (121, 122). Clinical studies have also shown that LAIV provides similarly high efficacy in both younger and older children, with no evidence that increasing levels of pre-existing influenza immunity diminish its effectiveness (123). After reformulation with updated strains and the accumulation of supportive clinical evidence, FluMist was reinstated in 2018 for use in healthy, non-pregnant individuals aged 2–49 years without contraindications, with a recommendation of “no preference” over other vaccine types (124–126). In September 2024, the U.S. Food and Drug Administration (FDA) further approved FluMist for self-administration (for individuals aged 18–49 years) or administration by caregivers aged ≥18 years (for children and adolescents aged 2–17 years) (127).

Co-administration of vaccines in children has mainly focused on meningococcal vaccines, diphtheria, tetanus, and acellular pertussis (DTaP)-containing combination vaccines, measles–mumps–rubella vaccines, and pneumococcal vaccines, while studies on co-administration with influenza vaccines are relatively limited (128). Currently, evidence shows that co-administration of LAIV with measles–mumps–rubella and varicella vaccines does not result in immune interference compared with separate administration, demonstrating comparable immunogenicity and good safety, and has been recommended by the U.S. CDC (129). Evidence on co-administration safety indicates that, in children aged 6 months to 2 years, simultaneous administration of IIV with pneumococcal conjugate vaccine (PCV13) or DTaP vaccines is associated with a slightly increased risk of febrile seizures within 24 hours; however, the overall incidence remains very low (up to 30 cases per 100,000 vaccinated children). The ACIP continues to recommend co-administration according to the immunization schedule, allowing influenza, pneumococcal, and DTaP vaccines to be administered during the same visit (130).

### 5.2 Adults

Adults generally possess a mature immune system. However, influenza infection in adults can facilitate transmission to more vulnerable populations, such as children and older adults. Therefore, vaccination of healthcare workers and caregivers is strongly recommended to reduce transmission to high-risk groups. WHO recommends annual influenza vaccination for individuals at increased risk, including healthcare workers, older adults, individuals with comorbidities, and pregnant women (131). For pregnant women, IIV or RIV may be used, whereas LAIV is contraindicated during pregnancy but may be administered postpartum (116, 132). For solid organ transplant recipients aged 18–64 years receiving immunosuppressive therapy, high-dose inactivated influenza vaccines (HD-IIV) or adjuvanted inactivated influenza vaccines (aIIV) may be considered to enhance the immune response (116). Strengthening vaccination campaigns and policies to improve adult vaccine uptake remains essential for increasing influenza vaccination coverage (133).

The RIV Flublok, despite its high antigen content (45 μg per strain per dose), has shown suboptimal hemagglutination inhibition (HI) antibody responses in clinical trials involving infants and young children (especially those aged 6–35 months and 3–8 years) (134), possibly due to the absence of additional immune stimulation (e.g., other viral antigens or adjuvants present in split vaccines) (135). Although studies indicate that Flublok provides safety and immunogenicity comparable to licensed IIV in individuals aged 9–17 years, it is currently approved by regulatory authorities only for use in individuals aged ≥18 years (136).

In adults, influenza VE typically ranges from 40% to 60%, depending largely on the antigenic match between circulating strains and vaccine strains (137). Even under conditions of mismatch due to antigenic drift, vaccines continue to confer partial protection, albeit with reduced efficacy. For example, IIV demonstrate VE of 52% (95% CI:37, 63) against mismatched influenza A strains, compared with 65% (95% CI:54,73) for matched strains (138). However, the substantial overlap in CI suggests that the impact of antigenic drift may be limited in some seasons, while larger antigenic changes can lead to more pronounced reductions in VE.

### 5.3 Older adults

Reduced VE and its accelerated decline in older adults are primarily attributed to immunosenescence, which progressively impairs both humoral and cellular immunity (139, 140). Thymic involution leads to reduced production of naïve T cells, decreased T cell diversity and function, and diminished responsiveness to novel influenza antigens arising from antigenic drift or shift (141).

Compared with younger adults (18–34 years), individuals aged ≥65 years exhibit impaired recall responses of pre-existing memory B cells and reduced plasmablast differentiation, resulting in shorter antibody durability (142). Many studies report that age-associated B cell defects lead to reduced antibody responses to influenza vaccines, with lower antibody titers observed in older adults compared to younger populations (143, 144). Aging impairs the differentiation and function of circulating CD4^+^ Tfh cells, thereby compromising GC maintenance by limiting the delivery of critical survival and differentiation signals, including IL-21 and CD40L, to B cells. Consequently, the generation and long-term maintenance of long-lived plasma cells and memory B cells are reduced, leading to accelerated waning of vaccine-induced antibody titers (145, 146). However, some studies have reported comparable or even higher post-vaccination antibody titers in older adults (147), possibly due to differences in baseline cytokine levels and pre-existing antibody titers. Notably, older adults tend to have a higher proportion of antibodies targeting conserved influenza epitopes, but these antibodies often have weaker neutralizing capacity, which may reduce VE (71, 148). In some cases, pre-existing immunity may have a greater impact on vaccine response than immunosenescence itself (149).

In parallel, cellular immune memory also undergoes accelerated functional decline with aging, limiting long-term protection when neutralizing antibodies wane or fail to recognize antigenically drifted viruses. Lung Trm, which provide rapid local antiviral protection, exhibit age-associated functional impairment and reduced protective capacity, thereby compromising early viral control (150). Circulating memory CD8^+^ T cells in older adults progressively acquire a senescent phenotype characterized by the accumulation of CD28^-^CD57^+^ cells, which exhibit reduced proliferative capacity, impaired recall responses, and diminished cytokine production (145, 151). Collectively, these defects compromise cross-reactive cellular immunity, thereby reducing protection against severe influenza, hospitalization, and death, particularly when circulating viruses are antigenically mismatched to vaccine strains.

Strategies to improve vaccine performance in older adults include increasing antigen dose (e.g., HD-IIV) or using adjuvanted formulations (e.g., adjuvanted influenza vaccines, aIIV) to enhance both humoral and cellular immune responses (152–154). ACIP recommends preferential use of HD-IIV or aIIV for adults aged ≥65 years to achieve stronger immune protection. Most European countries similarly recommend HD-IIV for individuals aged ≥60 or ≥65 years (155). A meta-analysis showed that, compared with standard-dose vaccines, HD-IIV induce higher geometric mean titers and seroprotection rates, and significantly reduce the risk of laboratory-confirmed influenza infection (relative risk 0.76) (156). Beyond use in individuals aged ≥60 years, HD-IIV is also being evaluated in phase III clinical trials in adults aged 50–64 years (NCT06641180). The MF59-adjuvanted influenza vaccine Fluad, based on a squalene oil-in-water emulsion system, was first licensed in Italy in 1997. According to the original Italian Medicines Agency (AIFA) marketing authorization (Decree No. 187, 23 April 1997). Historically, Fluad was initially administered to all individuals aged 12 years and above (157). Around 2000, Fluad became available in additional European countries through the Mutual Recognition Procedure (MRP) (158). By 2014, the AIFA described Fluad as being indicated for the active immunization of adults aged ≥65 years, especially those at increased risk of complications (159). More recently, in 2023, the European Medicines Agency (EMA) expanded the indication of the Fluad Tetra to individuals aged ≥50 years, reflecting increasing recognition that enhanced influenza vaccines may provide clinical benefits not only for older adults but also for middle-aged adults (50–64 years) (160).

VE in older adults typically ranges from 30% to 50%. According to interim estimates from the CDC for the 2023–2024 influenza season, VE against influenza-associated outpatient acute respiratory illness was 59%–67% in children and adolescents (6 months–17 years), 25%–52% in adults (18–64 years), and 41%–51% in older adults (≥65 years). VE against influenza-associated hospitalization was 52%–61%, 40%–49%, and 42%, respectively (161). Given the high prevalence of comorbidities and risk of secondary bacterial infections, co-administration of influenza and pneumococcal vaccines represents an effective strategy to reduce pneumonia incidence and all-cause mortality (162).

## 6 Conclusion and perspective

### 6.1 Application prospects of innovative vaccines in different populations

In recent years, influenza vaccine development has been shifting from a “one-dose-fits-all” approach toward “age-specific precision,” emphasizing the optimization of antigen design, adjuvant selection, and immunization strategies based on the immunological characteristics of different age groups. Infants, adults, and the elderly exhibit substantial differences in immune development, immune imprinting, and immunosenescence, providing a strong theoretical basis for differentiated vaccine strategies.

In children, preclinical and clinical studies suggest that adjuvanted vaccines can significantly enhance Tfh cell expansion and GC responses, thereby increasing antibody titers and promoting the establishment of memory B cell repertoires (163, 164). Adjuvanted influenza vaccines thus demonstrate strong application potential in children. The National Advisory Committee on Immunization (NACI) in Canada recommends the MF59-adjuvanted trivalent inactivated influenza vaccine (IIV3-Adj, Fluad Pediatric) for children aged 6–23 months at a dose of 0.25 mL (7.5 µg HA per strain) (165). In a phase III randomized multicenter trial conducted in Mexico among children aged 6 months to <72 months, MF59-adjuvanted trivalent influenza vaccine (Fluad^®^) elicited higher immune responses than non-adjuvanted influenza vaccine (Fluzone^®^), with a favorable safety profile (166). Additional studies indicate that, compared with non-adjuvanted IIV, the MF59-adjuvanted vaccine induces a dose-dependent enhancement of immune responses immediately after vaccination and sustains the HI antibody titers up to one year post-vaccination (167). A meta-analysis further showed that adjuvanted influenza vaccines exhibit superior immunogenicity in antigen-naïve children, whereas no significant difference is observed in non-naïve children (those previously vaccinated or infected) (168). During the 2009 influenza pandemic, monovalent H1N1 AS03-adjuvanted vaccines were widely used in children (164). These findings suggest that adjuvanted influenza vaccines are not only suitable for the elderly but are particularly beneficial for immunologically immature or primed-naïve populations, such as children aged 6–23 months, and may serve as an important strategy for optimizing primary immunization in children.

In adults, although the immune system is fully mature, prior infections or vaccinations may result in immune imprinting, potentially limiting cross-reactive responses to novel strains (169). “Immune reprogramming” strategies through optimized antigen design, sequential immunization (prime-boost), and conserved epitope targeting aim to induce broadly neutralizing antibodies and cross-subtype T cell responses (170, 171). Mucosal immunization strategies and novel delivery platforms (e.g., intranasal vaccines, nanoparticles, and microneedle patches)may partially circumvent systemic immune imprinting by inducing local immunity and activating Trm cells (172). Several innovative vaccine platforms have shown promising results in clinical studies in adults. For example, the adenoviral vector vaccine NasoVAX demonstrated that a single dose could induce robust humoral (serum IgG), cellular (T cell responses), and mucosal (IgA) immunity in healthy adults aged 18–49 years, with antibody responses lasting at least one year, suggesting potential for “single-dose long-term protection” (173). The modified vaccinia Ankara (MVA)-based T cell vaccine MVA-NP+M1 significantly reduced laboratory-confirmed influenza in human challenge studies (174). The intranasal M2-deficient single-replication (M2SR) influenza vaccine provided protection against highly drifted H3N2 strains and induced both mucosal and cellular immunity, conferring cross-protective antibodies in susceptible adults aged 18–49 years (175, 176), and has subsequently been evaluated for immunogenicity and safety in populations aged 65–85 years (NCT05163847). Nanoparticle vaccines such as FluMos-v1 and v2 present multiple HA antigens in a repetitive array to induce broadly neutralizing antibodies and are currently under clinical evaluation (NCT04896086, NCT06863142) (177). Multiple microneedle patch-based influenza vaccines are also being tested in healthy adults, showing comparable or enhanced immune responses relative to intramuscular vaccination (178, 179). However, most clinical validation has thus far focused on young adults, and further systematic evaluation is needed in populations with distinct immune profiles, such as children and the elderly.

In elderly populations, high-dose, adjuvanted, and immunomodulatory formulations have been shown to enhance immunogenicity (180). Combining these approaches with novel delivery systems or mucosal immunization strategies may enable synergistic systemic and local protection. Oil-in-water adjuvant systems such as AS03 have been successfully applied in pandemic influenza vaccines (e.g., Prepandrix and Pandemrix), inducing rapid and robust immune responses (181). Studies have shown that in individuals aged ≥65 years, AS03-adjuvanted vaccines induce higher HI antibody levels with improved durability (182). However, post-marketing surveillance linked Pandemrix to an increased risk of narcolepsy in children and adolescents, highlighting the importance of formulation-specific safety evaluation (183). Following the end of the 2009 influenza pandemic, Pandemrix was discontinued in Europe, while the association between vaccination and narcolepsy was investigated by the marketing authorisation holder (184). Next-generation adjuvants designed to stimulate multiple innate immune pathways—includingTLR3, TLR7/8, STING, or RIG-I agonists, as well as combination adjuvant systems—are being investigated to overcome immunosenescence (185–188). TLR7/8 agonist adjuvant, or combination adjuvant, can enhance GC formation, Th1-biased cellular immunity, and cross-reactive antibody responses in aged mice (187, 189). Furthermore, combining a STING activator with a saponin-based adjuvant facilitates intracellular delivery of exogenous cGAMP and enhances STING pathway activation, thereby improving vaccine-induced immune responses in aged mice (185). The Matrix-M–adjuvanted nanoparticle influenza vaccine (NanoFlu™) has been shown to induce cross-reactive HI antibodies and multifunctional CD4^+^ T cell responses in individuals aged ≥65 years in clinical studies (190, 191).

Computationally optimized broadly reactive antigen (COBRA)-based immunogens can induce broad cross-reactive antibody responses and provide enhanced protection against antigenically divergent influenza viruses, supporting their potential application in next-generation vaccines for elderly populations (192). In addition, heterologous antigen exposure strategies using sequential presentation of distinct but related influenza antigens may broaden T-cell help, enhance immune repertoire diversity, and improve functional immune memory (193). Structure-based immunogen designs focusing on conserved influenza epitopes, particularly the HA stem region, provide complementary approaches by redirecting immune responses toward conserved targets and reducing vulnerability to antigenic drift (194, 195). Intranasal multivalent nanoparticle-based influenza vaccines have been reported to increase mucosal antibody responses, induce cross-neutralizing antibodies, and enhance influenza-specific CD4^+^ and CD8^+^ T-cell responses (196). Given the age-related decline in antibody responses, strategies designed to strengthen conserved influenza-specific T-cell immunity may provide an important complementary approach for older adults by overcoming limitations associated with reduced T-cell repertoire diversity and impaired immune memory formation (197). Clinical trials of MVA-NP+M1 in elderly populations indicate that it primarily enhances cellular immunity and can be co-administered with traditional inactivated vaccines to improve overall protection (198). Such approaches may complement antibody-focused strategies and help overcome age-related impairments in T-cell repertoire diversity and immune memory formation.

mRNA technology has emerged as one of the most promising next-generation platforms for influenza vaccine development. In phase III clinical trials, mRNA-1010 met the primary non-inferiority immunogenicity endpoints at Day 29 compared with both the licensed standard-dose IIV (Fluarix^®^) and the HD-IIV (Fluzone High-Dose). In adults aged ≥65 years, prespecified superiority criteria were met for all four influenza strains based on Day 29 HI geometric mean ratio (GMR) (97.5%/98.8% CI lower bounds >1) and seroconversion rate (SCR) differences (97.5%/98.8% CI lower bounds >0%), with a higher frequency of solicited local adverse reactions than comparator vaccines (199). Furthermore, in a phase III efficacy trial involving adults aged ≥50 years, mRNA-1010 achieved the most stringent prespecified superiority criterion, with a relative vaccine efficacy (rVE) of 26.6% (95% CI: 16.7, 35.4) compared with standard-dose Fluarix^®^. Compared with Fluad in adults aged 18-75 years, mRNA-1010 induced higher H3-specific memory B-cell responses together with comparable CD4^+^ and a trend toward stronger CD8^+^ T-cell responses (200). Although mRNA-1010 has been submitted for regulatory approval, no head-to-head efficacy trial has yet compared mRNA-1010 with enhanced influenza vaccines, including HD-IIV or aIIV, which represent the current age-appropriate standard-of-care comparators for older adults in regulatory evaluation (201). Importantly, the successful global deployment of mRNA-based COVID-19 vaccines has demonstrated the applicability of the mRNA platform across the entire lifespan. Licensed vaccines such as Comirnaty^®^ and Spikevax^®^ have been authorized for use in individuals aged ≥6 months and have shown robust immunogenicity in children, adults, and older adults (202, 203). These experiences indicate that the value of mRNA technology extends beyond addressing immunosenescence in elderly populations. Through optimization of antigen design, mRNA dose, lipid nanoparticle formulations, and immunization schedules, mRNA influenza vaccines could potentially address distinct age-related immunological challenges, including immune development in children, immune imprinting in adults, and immunosenescence in older adults.

Beyond vaccine platform optimization, strategies aimed at directly counteracting immunosenescence may further improve influenza vaccine efficacy in older adults. Interleukin-7 (IL-7), a key regulator of T-cell homeostasis, promotes thymopoiesis, naïve T-cell maintenance, and T-cell receptor diversity. IL-7 agonists have been shown to enhance antigen-specific CD4^+^ and CD8^+^ T-cell responses and may serve as vaccine immunopotentiators in elderly individuals (204). In addition, senolytic approaches targeting accumulated senescent cells may alleviate inflammaging and restore immune responsiveness (205, 206), whereas trained immunity strategies aim to enhance innate immune memory through epigenetic and metabolic reprogramming (207). Although these approaches remain largely preclinical, they provide potential avenues for overcoming age-related immune dysfunction.

### 6.2 Challenges and limitations

Despite substantial progress in influenza vaccinology, several major challenges remain. First, the immunological mechanisms underlying vaccine responsiveness across different age groups are still incompletely understood. Although immune development, immune imprinting, and immunosenescence have emerged as key determinants, the precise interactions among host genetics, prior exposure history, mucosal immunity, and inflammatory regulation require further investigation (72, 208). Second, the long-term effectiveness and feasibility of innovative vaccine platforms remain to be fully validated. While mRNA vaccines, nanoparticle vaccines, viral vectors, and novel adjuvant systems have demonstrated encouraging early clinical results, questions remain regarding durability of protection, scalability of manufacturing, storage stability, regulatory feasibility, cost-effectiveness, and long-term safety across diverse populations (209). Third, most current vaccine evaluation systems primarily rely on HI antibody titers as correlates of protection; however, HI provides only a partial assessment of protective immunity. HI mainly reflects HA head-directed antibodies and does not adequately capture HA stalk antibodies, mucosal IgA responses, NA-specific antibodies, Fc-mediated functions, or T-cell immunity. Therefore, for next-generation influenza vaccines, including universal vaccines, nanoparticle vaccines, and T-cell-focused platforms, multidimensional immune correlates integrating humoral, mucosal, and cellular responses will be required to better predict vaccine efficacy and mechanisms of protection (210–212). Finally, the implementation of age-stratified vaccination strategies presents important practical and regulatory challenges. Developing individualized or population-specific vaccines may increase manufacturing complexity, regulatory burden, and global distribution inequities. Whether a single adaptable platform can be engineered to generate distinct immune outcomes in different populations remains uncertain and represents a major scientific challenge for next-generation vaccine development (209).

### 6.3 Conclusion

In conclusion, age-dependent immune heterogeneity plays a central role in shaping influenza pathogenesis and vaccine responsiveness throughout life. Infants and children exhibit immune development and limited immune memory, adults are strongly influenced by immune imprinting, and older adults experience progressive immunosenescence and inflammaging. These distinct immune landscapes provide the immunological foundation for precision influenza vaccination strategies.

Future influenza prevention should move beyond uniform vaccination approaches toward integrated, age-adapted immunization frameworks incorporating innovative vaccine platforms, optimized antigen design, advanced adjuvant systems, and mucosal immunization technologies. Achieving this goal will require a transition from empirical vaccine improvement toward mechanism-guided vaccine design, with a better understanding of how age-specific immune pathways regulate vaccine responses. Integration of single-cell transcriptomics, systems vaccinology, computational approaches, and comprehensive immune profiling may identify predictive biomarkers of vaccine responsiveness and enable optimization of vaccine formulation, antigen selection, and immunization schedules. Such approaches may also help clarify why individuals with distinct immune aging profiles, particularly older adults, exhibit variable vaccine responses and support the development of personalized vaccination strategies. Through continued advances in immunology, vaccinology, translational medicine, and global collaboration, next-generation influenza vaccines may ultimately achieve broader, more durable, and more equitable protection across all age groups.
